# Supplementary material for: Differential conformational expansion of NUP98-HOXA9 oncoprotein from nanosized assemblies to macrophases
Source: Nat Commun. 2025 Nov 18;16:10117. doi: 10.1038/s41467-025-66327-1 (PMC12627784; doi:10.1038/s41467-025-66327-1)
Supplement: Supplementary file 1 — Supplementary Information [file 41467_2025_66327_MOESM1_ESM.pdf]

Supplementary information

Differential conformational expansion of NUP98-HOXA9 oncoprotein from nanosized assemblies to macrophases

Hao Ruan[1,2#], Rodrigo F. Dillenburg[3#], Elnaz Hosseini[4], Sina Wittmann[4,5], Martin Girard[3,5*], Edward A. Lemke[1,4*]

[1]Biocenter, Johannes Gutenberg University Mainz, Mainz 55128, Germany.

[2]Institute of Molecular Biology postdoctoral program, Mainz 55128, Germany.

[3]Max-Planck Institute for Polymer Research, Mainz 55128, Germany.

[4]Institute of Molecular Biology (IMB gGmbH), Mainz 55128, Germany.

[5]Institute for quantitative and computational biosciences, Mainz 55128, Germany.

[#]These authors contributed equally: Hao Ruan, Rodrigo F. Dillenburg.

*Corresponding authors:

Martin Girard

Email: martin.girard@mpip-mainz.mpg.de

Edward A. Lemke

Email: edlemke@uni-mainz.de

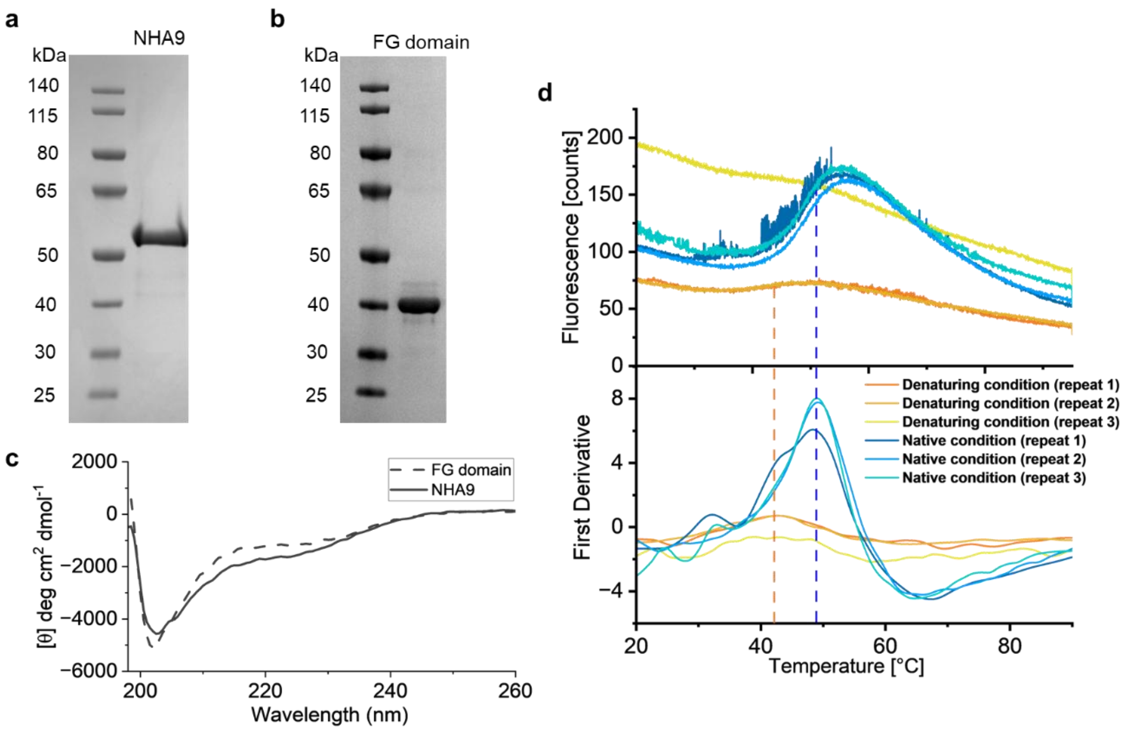

Supplementary Figure 1. Characterization of purified NHA9 and the FG domain. a,b SDS-PAGE analysis of purified NHA9 and FG domain alongside molecular mass markers. **c** CD spectra of NHA9 (solid line) and the FG domain (dash line). NHA9 exhibited a stronger signal at 222 nm ($[\theta]_{222}$ = –1630 deg·cm²·dmol⁻¹) compared to the FG domain ($[\theta]_{222}$ = –1171 deg·cm²·dmol⁻¹), whereas at 200 nm the FG domain showed a stronger signal ($[\theta]_{200}$ = –3059 deg·cm²·dmol⁻¹) relative to NHA9 ($[\theta]_{200}$ = –2732 deg·cm²·dmol⁻¹). These results suggest the presence of α-helical structures in the NHA9 sample. **d** NanoDSF measurements of NHA9. Under denaturing condition (yellow), NHA9 displayed a melting temperature (T_m) of 42.7 ̊C, whereas under native conditions, the T_m was 48.8 ̊C.

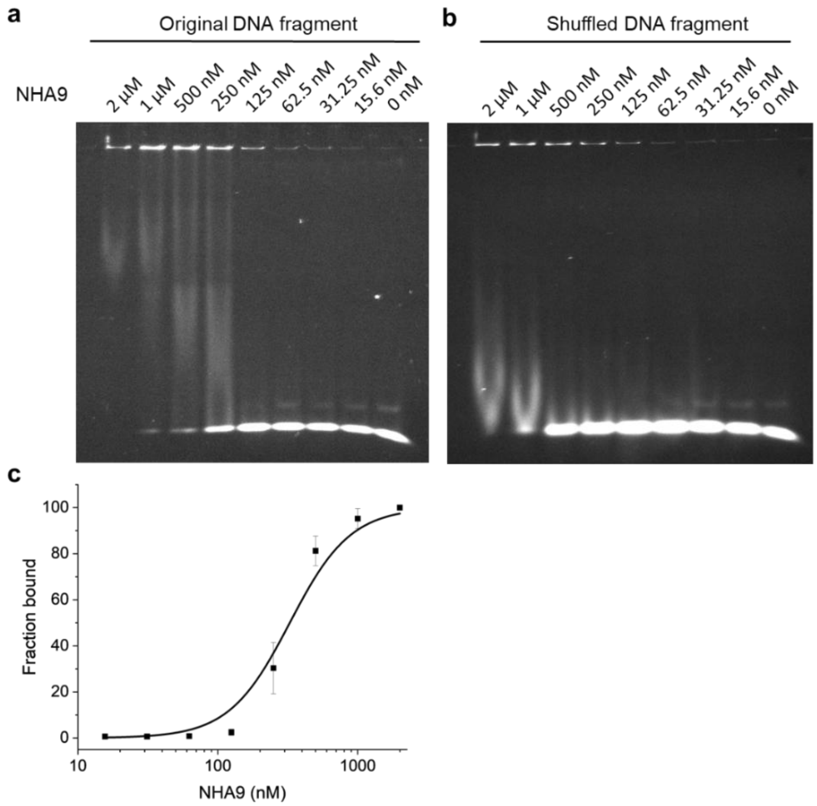

Supplementary Figure 2. DNA-binding ability of NHA9 assessed by Gel mobility shift assay (EMSA). **a** EMSA between NHA9 and original DNA fragment with HOXA9 binding site. **b** EMSA of NHA9 with a shuffled DNA fragment. **c** The binding affinity curve was fitted using band intensities from panel (a), derived from three replicates, yielding a K_D of 0.3 μM. The binding affinity for the shuffled DNA is too low to be extracted from this gel analysis.

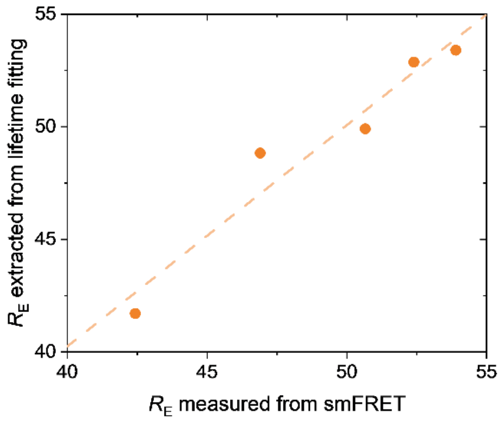

Supplementary Figure 3. Correlation between inter-residue distance R_E measured by intensity-based single-molecule FRET and those obtained from lifetime-based fitting. The red dotted line indicates the fitted correlation curve. The high correlation coefficient (R^2 = 0.95) validates the accuracy and reliability of our FLIM-FRET pipeline for measuring R_E.

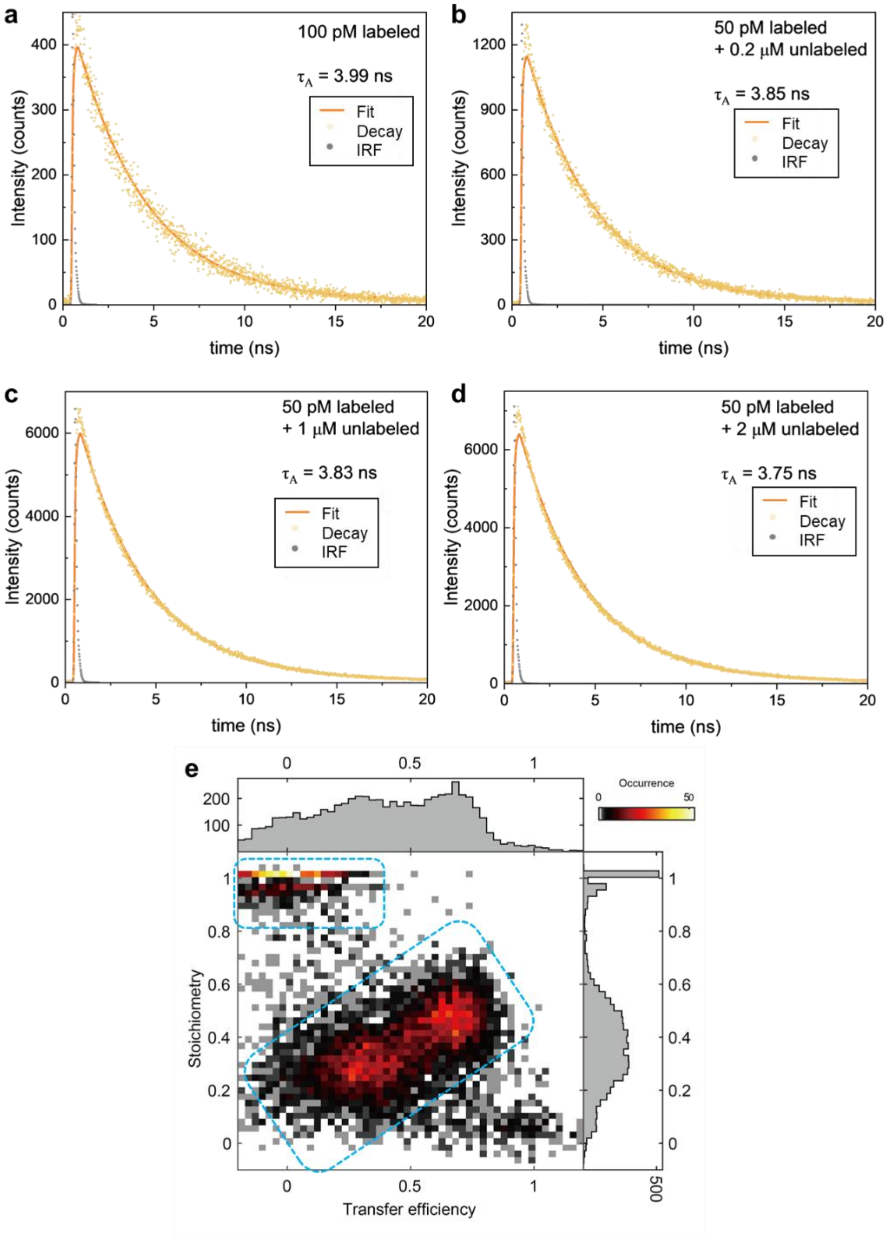

Supplementary Figure 4. Fluorescence lifetime decays and fitting of Alexa 594 acceptor conjugated to NHA9 at different concentrations and a 2D histogram of stoichiometry versus transfer efficiency values. Lifetime decays were obtained from single-molecule FRET measurements during the acceptor excitation cycle. **a** The fluorescence lifetime of Alexa 594 conjugated to NHA9 in the monomeric state was 3.99 ns. Increasing NHA9 concentrations resulted in decreased lifetimes: **b** 3.85 ns at 0.2 μM, **c** 3.83 ns at 1 μM, and **d** 3.75 ns at 2 μM NHA9. These data indicate that the acceptor undergoes fluorescence quenching within nanoclusters. **e** The 2D histogram of the stoichiometry values versus the mean transfer efficiency $\langle E \rangle$ for $NHA9_{A221\text{-}S362}$ at 1 μM concentration. The Donor-only species and FRET species, highlighted by blue rectangles, were individually selected for separate fitting using a 2D Gaussian function. The change in stoichiometry position indicates a shift in gamma between monomer and nanocluster. This is accounted for using the lifetime-based analysis.

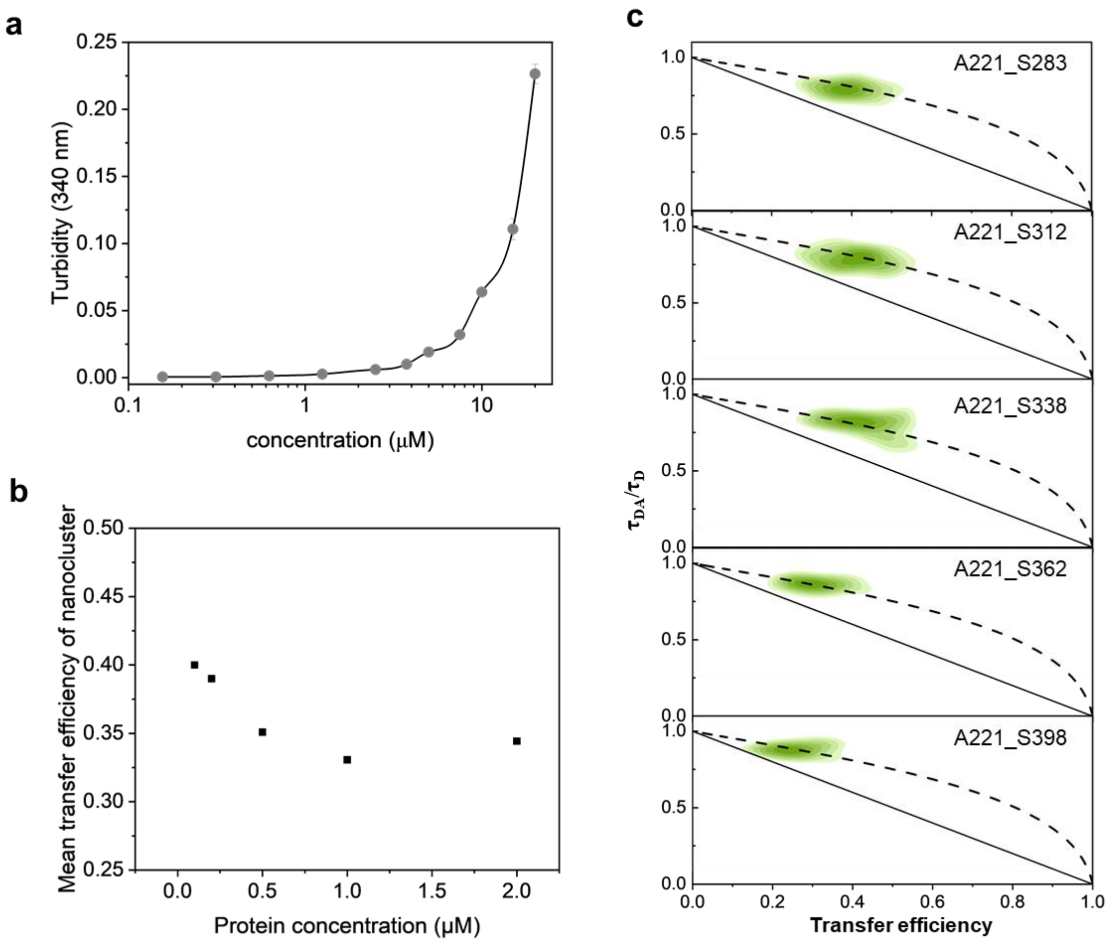

Supplementary Figure 5. Turbidity assay and nanocluster analysis by smFRET. **a** concentration-dependent turbidity of purified NHA9, measured by UV absorbance at 340 nm from three replicates. **b** Mean transfer efficiency extracted from single-molecule FRET measurements analog to main Figure 2b of NHA9 nanocluster at varying concentrations. **c** Two-dimensional histogram of the relative donor fluorescence lifetime (τ_{DA}/τ_{D}) versus mean transfer efficiency $\langle E \rangle$ calculated from individual bursts of five nanocluster variants at 1 μM. The dashed line indicates the dynamic relationship predicted by a Gaussian chain polymer model. See Methods in maintext for additional details.

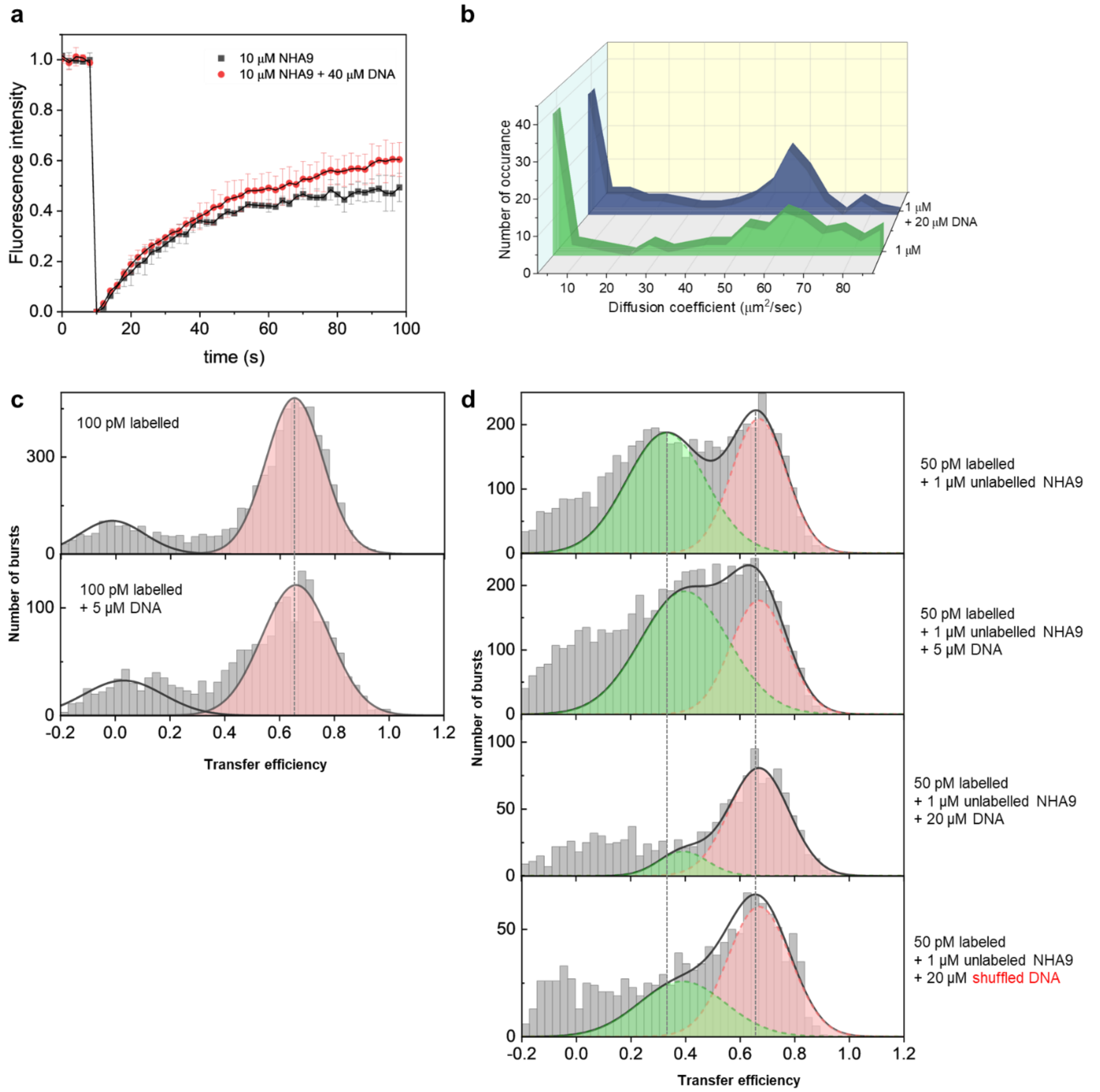

Supplementary Figure 6. Effects of DNA fragments on NHA9 condensate and nanocluster formation. **a** FRAP recovery curves of NHA9 condensates in the presence and absence of DNA fragment. Data are presented as mean ± SD (n = 3). **b** Nanocluster formation of NHA9 with or without DNA fragment at 1 μM, assessed by FCS. Autocorrelation curves were derived from 120 repeated measurements with short sampling time (10 s), using $NHA9_{A221C}$ labeled with LD655 as fluorescent probe in trace amount (10 nM) with excess unlabeled NHA9. **c** Single-molecule transfer efficiency histograms of $NHA9_{A221\text{-}S362}$ at 100 pM with or without 5 μM original DNA fragments. **d** Single-molecule transfer efficiency histograms of $NHA9_{A221\text{-}S362}$ at 1 μM unlabeled protein with varying concentrations of DNA fragments. The shuffled DNA fragment only partially inhibited NHA9 nanocluster formation, suggesting the coexistence of specific and non-specific interactions between NHA9 and the double-stranded recognition DNA fragment.

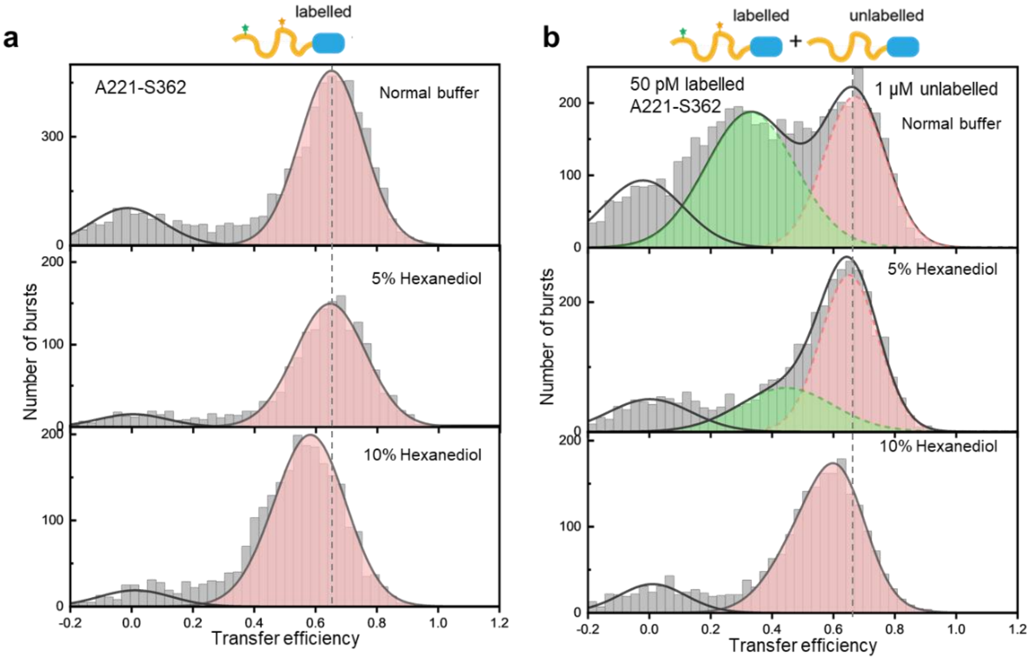

Supplementary Figure 7. Effects of 1,6-hexanediol NHA9 and nanocluster formation. a Single-molecule transfer efficiency histograms of $NHA9_{A221\text{-}S362}$ at 50 pM in native buffer, in native buffer containing 5% 1,6-hexanediol and 10% 1,6-hexanediol, respectively. **b** Single-molecule transfer efficiency histograms of $NHA9_{A221\text{-}S362}$ at 1 µM unlabelled protein under native buffer, native buffer containing 5% 1,6-hexanediol and 10% 1,6-hexanediol, respectively.

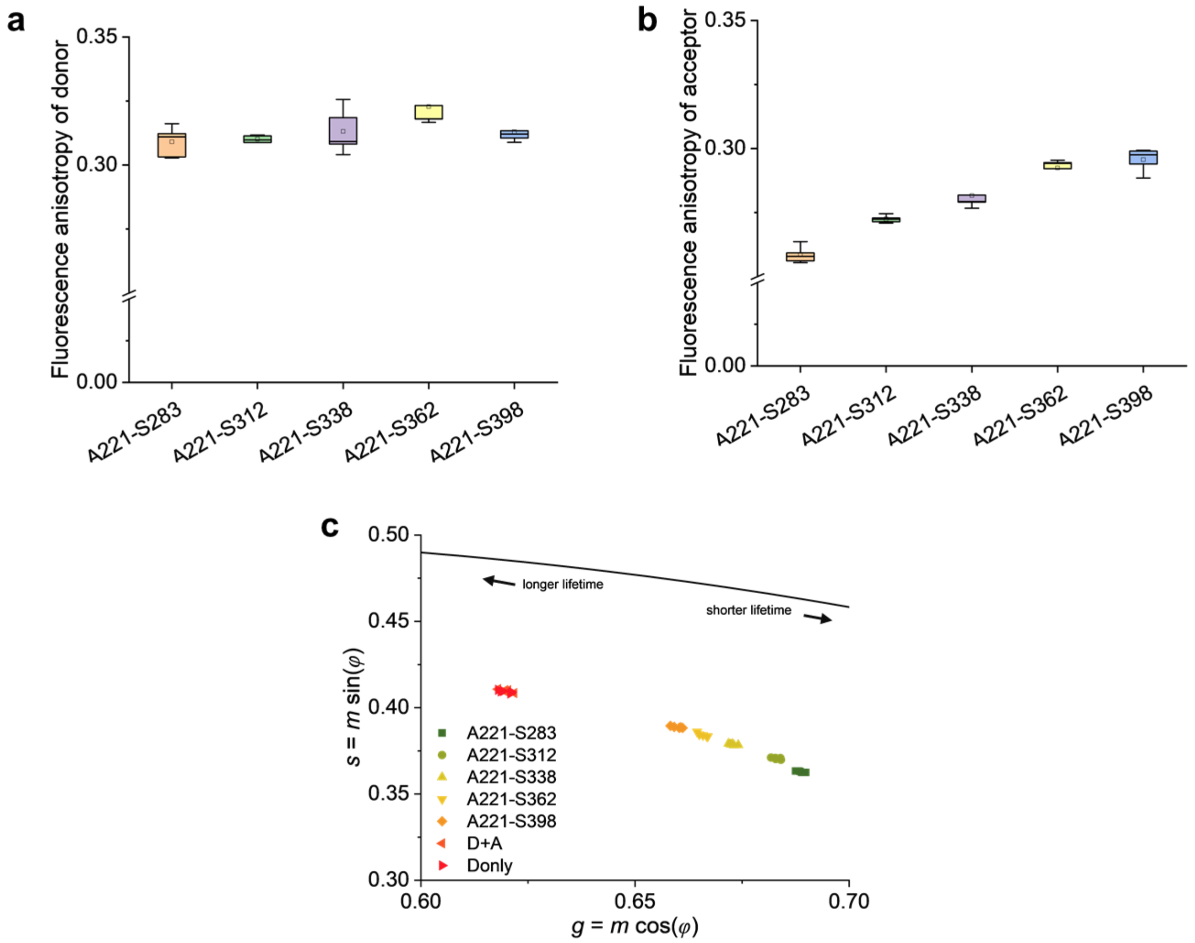

Supplementary Figure 8. FLIM-FRET analysis of NHA9 condensates *in vitro*. NHA9 variants were site-specifically labelled with a FRET dye pair, with 1 nM labeled protein mixed with 10 µM unlabelled protein. Each variant was analyzed in 5 replicates. **a** Box plot of fluorescence anisotropy values of the donor dye at different labeling sites in NHA9 condensates. **b** Box plot of fluorescence anisotropy values of the acceptor dye at different labeling sites in NHA9 condensates. Donor dye anisotropy values exceeded 0.3 across all sites, while acceptor dye anisotropy values remained below 0.3. In this regime, the error in distance measurements is estimated to be less than 10%.[1] **c** Phasor plots of donor fluorescence signals from NHA9 variants in *in vitro* reconstituted condensates. Each sample was measured in 5 repeats. No differences were observed between donor-only and donor–acceptor labeled single mutants, indicating the absence of intermolecular FRET events. The black universal circle represents the phasor plot of a single decay component with different lifetime. In box plots, the center line represents the median, the tiny square correspond to the mean, the box edges correspond to the 25th and 75th percentiles, and the whiskers extend to the minimum and maximum values within 1.5× the interquartile range.

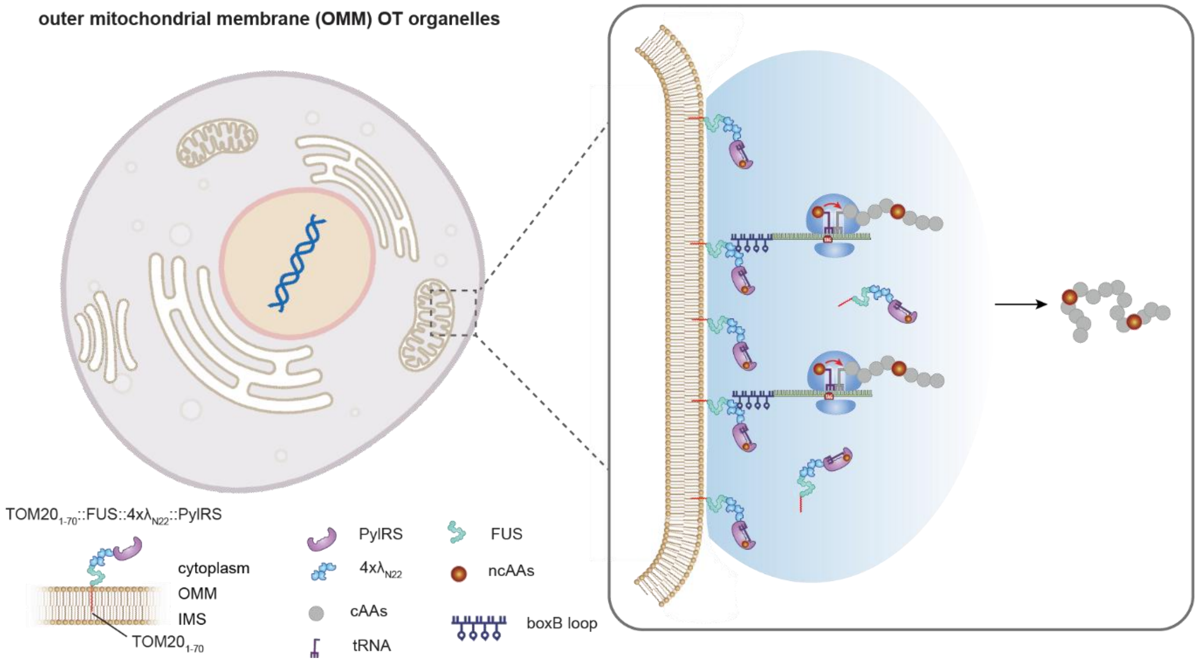

Supplementary Figure 9. Schematic of NHA9 labeling by outer mitochondrial membrane OTO-GCE system. PylRS and λ_{N22} were fused to the phase-separating scaffold FUS and the mitochondrial outer membrane-targeting domain TOM20$_{1-70}$. NHA9 mRNA was engineered with boxB RNA hairpin loops, which specifically bind to the λ_{N22} peptides. This design enabled selective recruitment of NHA9 mRNA into orthogonally translating organelle (OTO) system, thereby restricting the incorporation of non-canonical amino acids (ncAAs) to NHA9 and preventing their incorporation into other proteins[2,3,4].cAAs: canonical amino acids. IMS: inner mitochondrial membrane.

Created in BioRender. Lemke, E. (2025) https://BioRender.com/sybcnrf

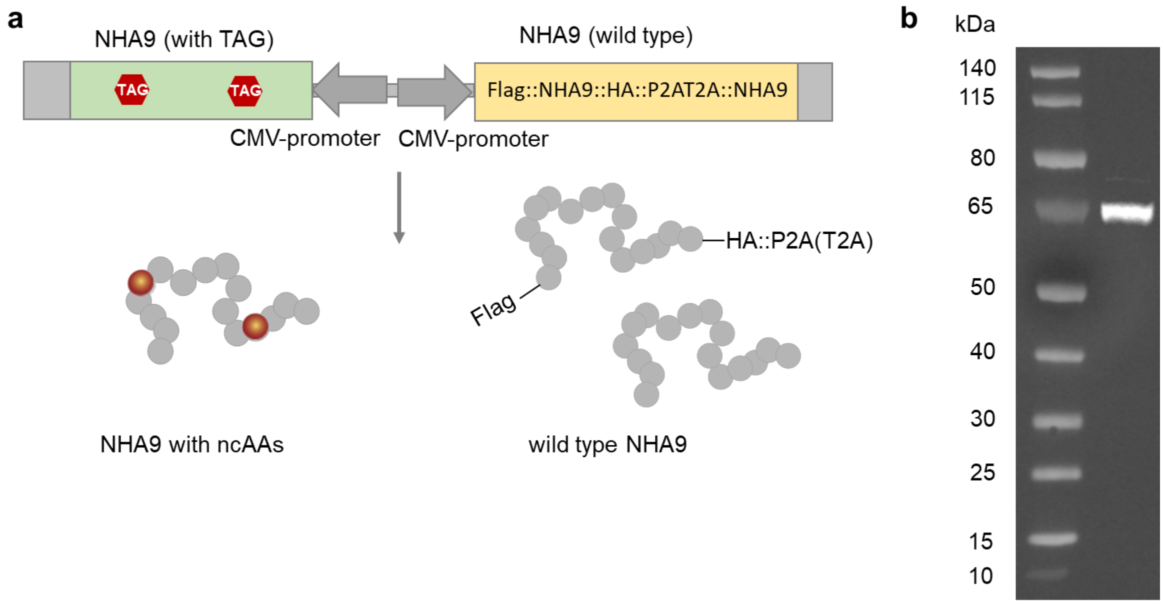

Supplementary Figure 10. NHA9 expression in HeLa cells. a Schematic representation of constructs used for NHA9 expression. To account for the heterogeneity of transient transfection, the construct was designed to co-express both wild-type NHA9 and TAG-mutant NHA9 within the same cells. **b** Western blot analysis of wild-type NHA9 expression using an anti-HA antibody. Due to ribosomal skipping induced by 2A peptides, cells expressed either Flag-NHA9-HA-P2A-T2A (65.8 kDa) or Flag-NHA9-HA-P2A (63.8 kDa), depending on the specific site of skipping.

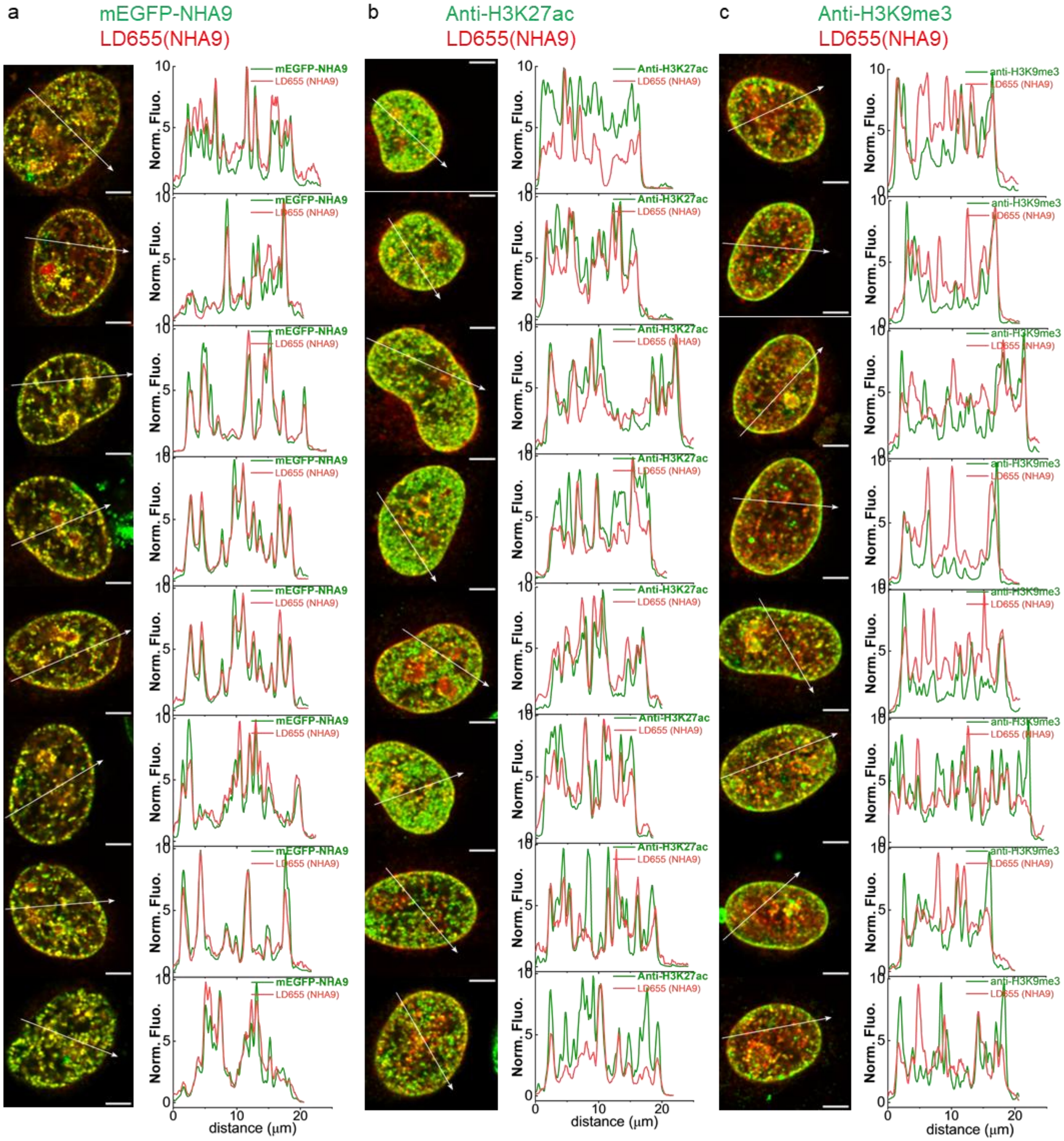

Supplementary Figure 11. Colocalization analysis between orthogonally translating organelles (OTOs) labelled with NHA9 and different targets. a Colocalization between OTOs labelled with NHA9 and mEGFP–NHA9 fusion. **b** Colocalization between OTOs labelled with NHA9 and H3K27ac. **c** Colocalization between OTOs labelled with NHA9 and H3K9me3. Scale bar: 5 μm.

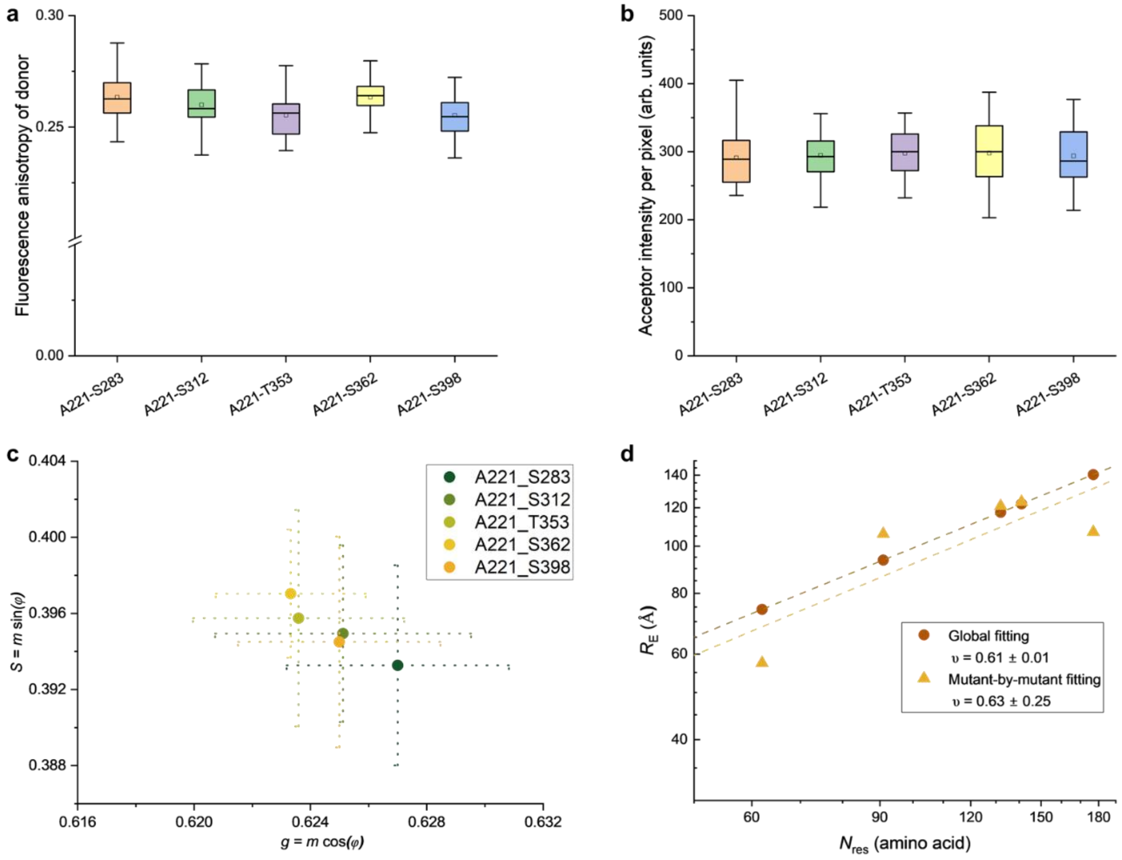

Supplementary Figure 12. Analysis of FLIM-FRET data in cells. a Box plot of donor anisotropy measurements across different NHA9 variants. All anisotropy values were below 0.3, indicating sufficient rotational mobility for FRET measurements of the donor fluorophore in the cellular environment. **b** Box plot of acceptor fluorescence intensity per pixel within nuclear condensates under 660-nm laser excitation. All five variants exhibited similar expression levels, with no cells displaying abnormally high expression included in the dataset. **c** Phasor plots of fluorescence donor channel signals for NHA9 variants. **d** Comparison of the R_E obtained from global fitting versus mutant-by-mutant fitting using the Gaussian chain model. In global fitting, fluorescence lifetime decays from all variants were simultaneously fitting using Eq. 6 to extract the scaling exponent across all measured cells. In mutant-by-mutant fitting, R_E values were first individually extracted for each variant by fitting its respective lifetime decay using Eq. 9, and the scaling exponent was then determined by extracting R_E versus N_{res} using Eq. 6. v is presented as the fitted value ± the standard error of the estimate. The cell counts for FLIM-FRET measurements of the A221-S283, A221-S312, A221-T353, A221-S362 and A221-S398 variants were 32, 29, 16, 44 and 42, respectively. In box plots, the center line represents the median, the tiny square correspond to the mean, the box edges correspond to the 25th and 75th percentiles, and the whiskers extend to the minimum and maximum values within 1.5× the interquartile range.

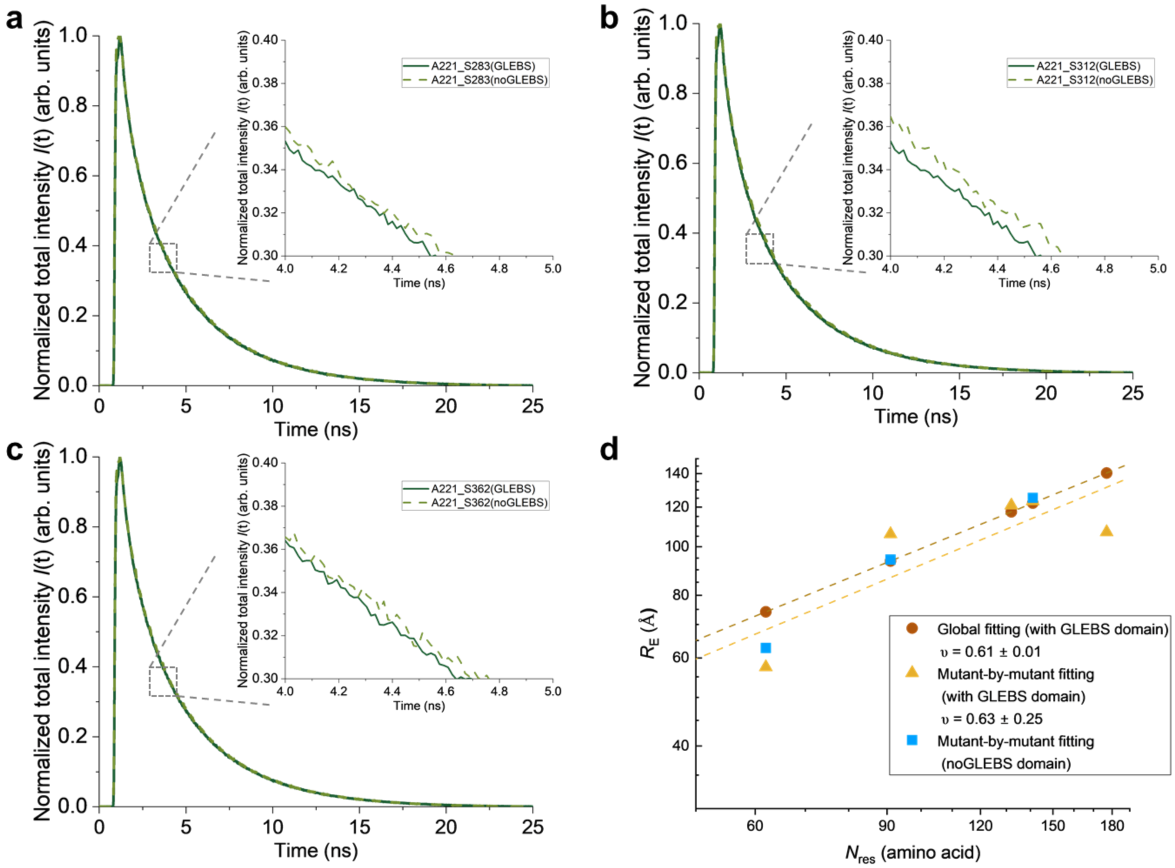

Supplementary Figure 13. Comparison of FLIM–FRET measurements between NHA9 constructs with and without the GLEBS domain. Normalized donor fluorescence decays of NHA9 variants A221–S283 (**a**), A221–S312 (**b**), and A221–S362 (**c**) with and without the GLEBS domain in cells. **d** Comparison of R_E values obtained from mutant-by-mutant fitting of NHA9 variants with and without the GLEBS domain. v is presented as the fitted value ± the standard error of the estimate.

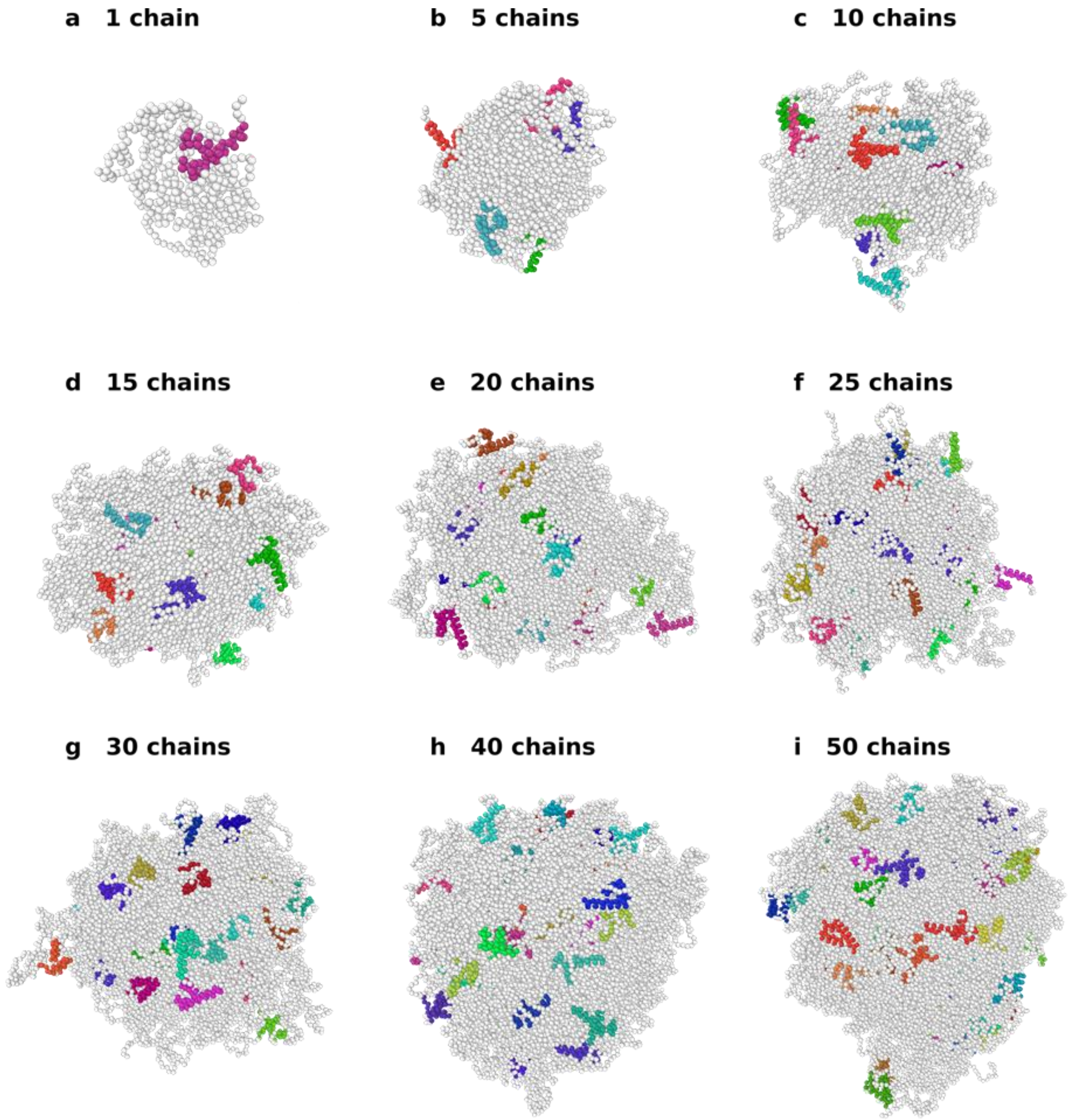

Supplementary Figure 14. Snapshots of clusters of different sizes. The 3 alpha-helices making up the (DNA-binding domain) DBD are colored, different colors represent different protein chains. Snapshots were made with OVITO[5].

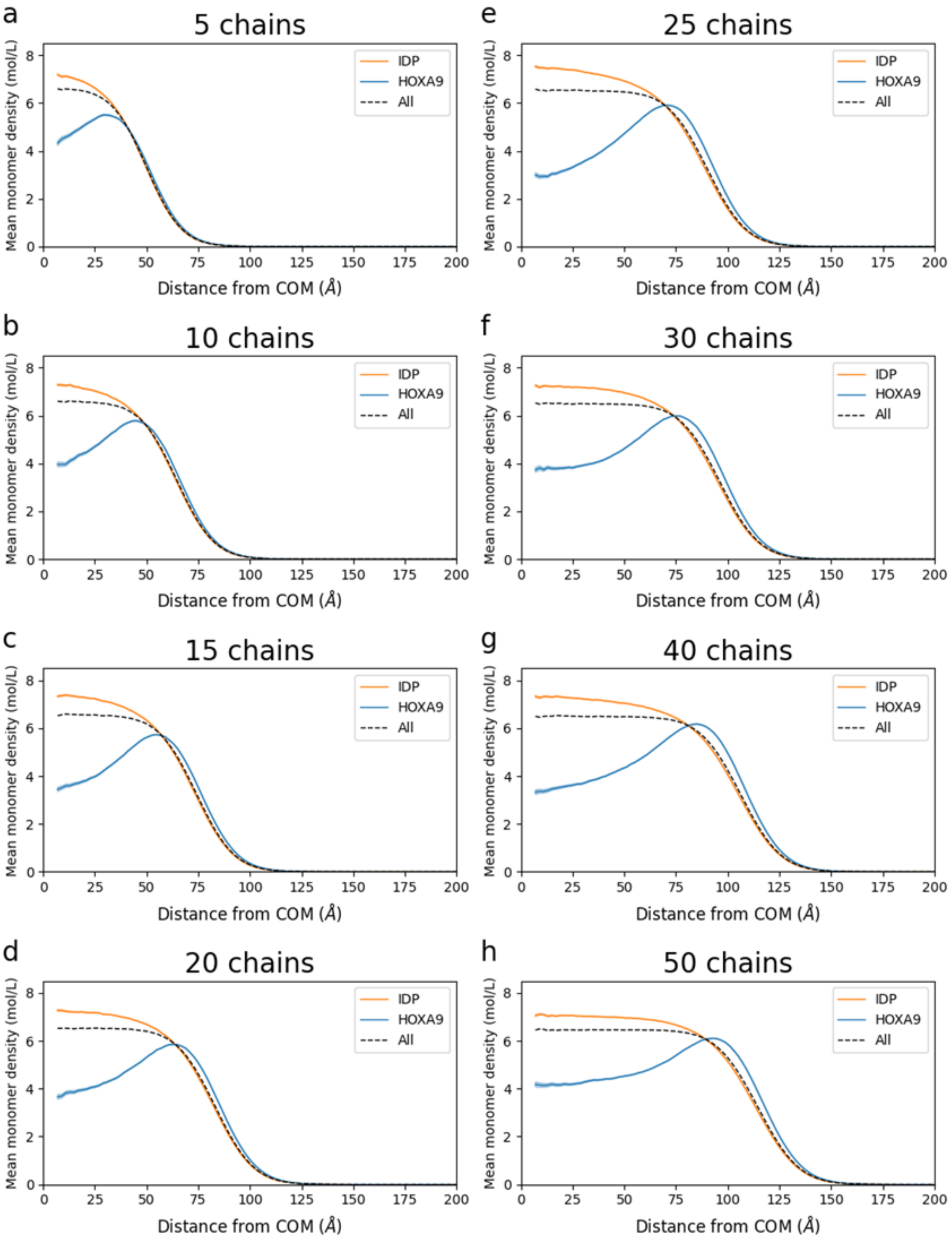

Supplementary Figure 15. Density profiles for individual clusters. Orange lines represent the intrinsically disordered protein (IDP) block, blue lines represent the HOXA9 block and the dashed black lines correspond to full-length NHA9. Units are moles of amino acids per liter. Data are presented as mean values ± standard error of the mean (SEM). Error bars are shown as shaded regions, which in most cases are too small to be visible. The number of statistically independent samples for each point on the x-axis corresponds to the number of frames in the production run, listed in the last column of Supplementary Table 3. Source data are provided as a Source Data file.

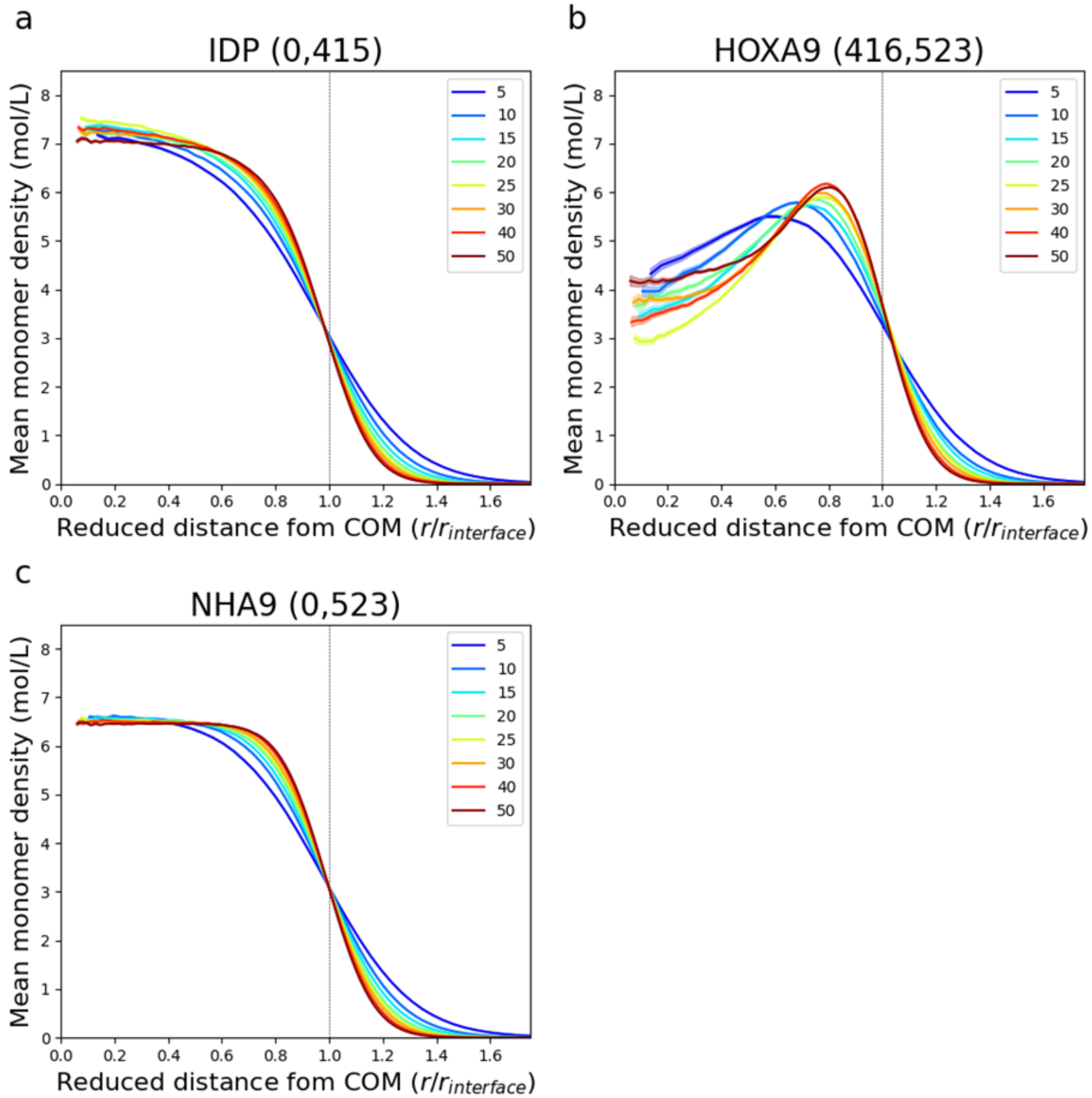

Supplementary Figure 16. Density profiles for both blocks of NHA9. a IDP block, **b** HOXA9 block and **c** full protein. Units are moles of amino acids per liter. Colors indicate clusters of different sizes. The dashed black line represents the center of the cluster's interface. The numbers next to the block names indicate the indices of the first and last amino acid in that block. Data are presented as mean values ± standard error of the mean (SEM). Error bars are shown as shaded regions, which in most cases are too small to be visible. The number of statistically independent samples for each point on the x-axis corresponds to the number of frames in the production run, listed in the last column of Supplementary Table 3. Source data are provided as a Source Data file.

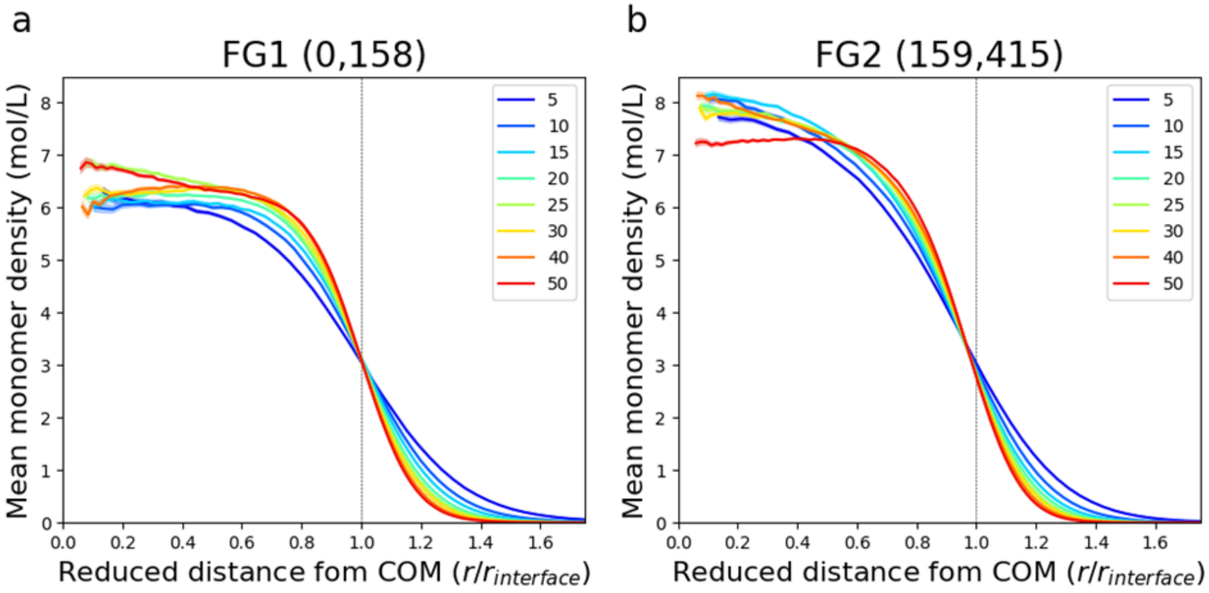

Supplementary Figure 17. Density profiles with the IDP divided into two blocks. a FG1 block, **b** FG2 block. The FG1 region includes the chain end of the intrinsically disordered protein (IDP) block. Units are moles of amino acids per liter. Colors indicate clusters of different sizes. The dashed black line represents the center of the cluster's interface. The numbers next to the block names indicate the indices of the first and last amino acid in that block. Data are presented as mean values ± standard error of the mean (SEM). Error bars are shown as shaded regions, which in most cases are too small to be visible. The number of statistically independent samples for each point on the x-axis corresponds to the number of frames in the production run, listed in the last column of Supplementary Table 3. Source data are provided as a Source Data file.

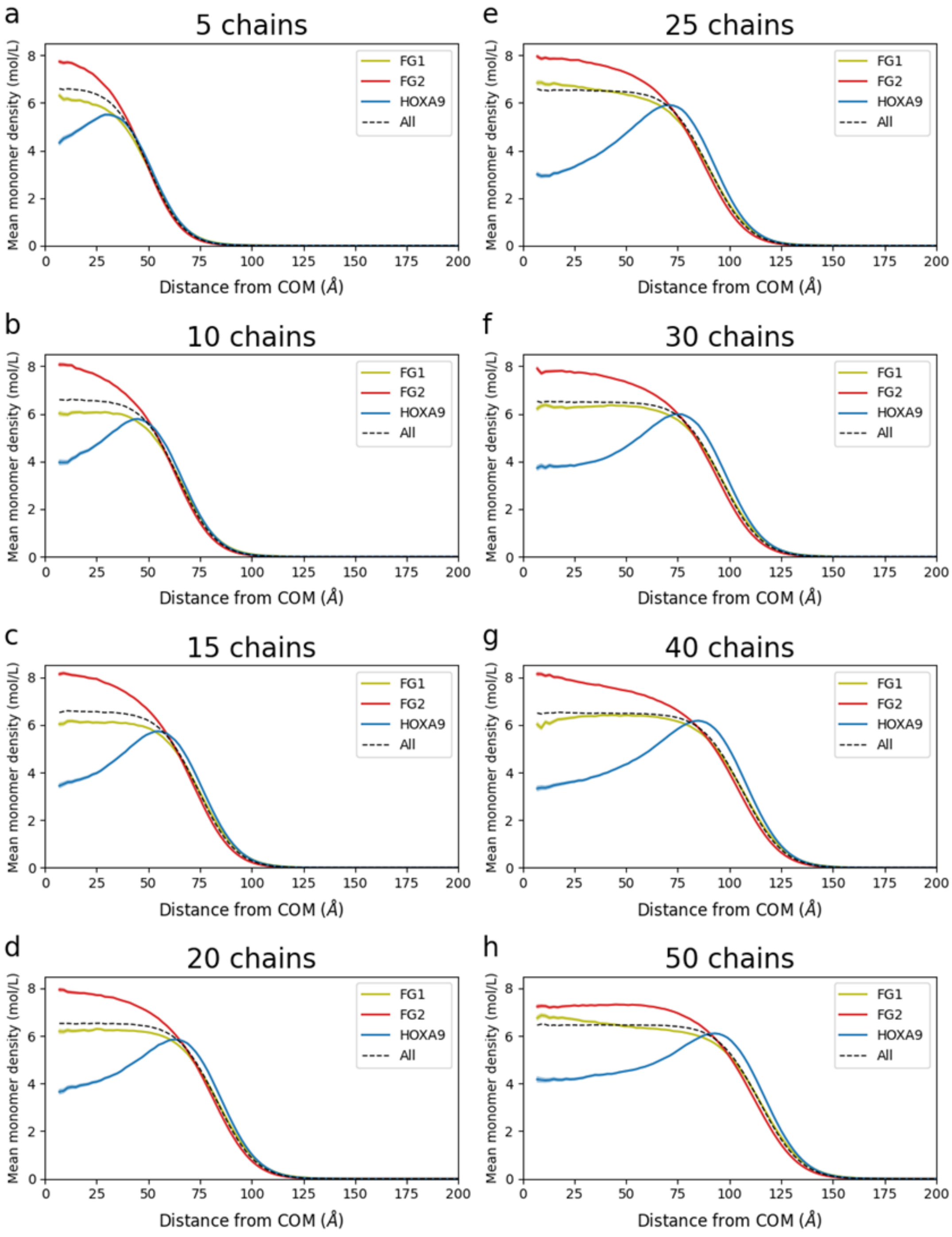

Supplementary Figure 18. Density profiles of individual clusters. Red and olive lines represent the FG1 and FG2 regions of the intrinsically disordered protein (IDP) block, respectively. Blue lines represent HOXA9, and the dashed black lines correspond to full-length NHA9. Units are moles of amino acids per liter. FG1 is located at the chain end of the IDP block. Data are presented as mean values ± standard error of the mean (SEM). Error bars are shown as shaded regions, which in most cases are too small to be visible. The number of statistically independent samples for each point on the x-axis corresponds to the number of

frames in the production run, listed in the last column of Supplementary Table 3. Source data are provided as a Source Data file.

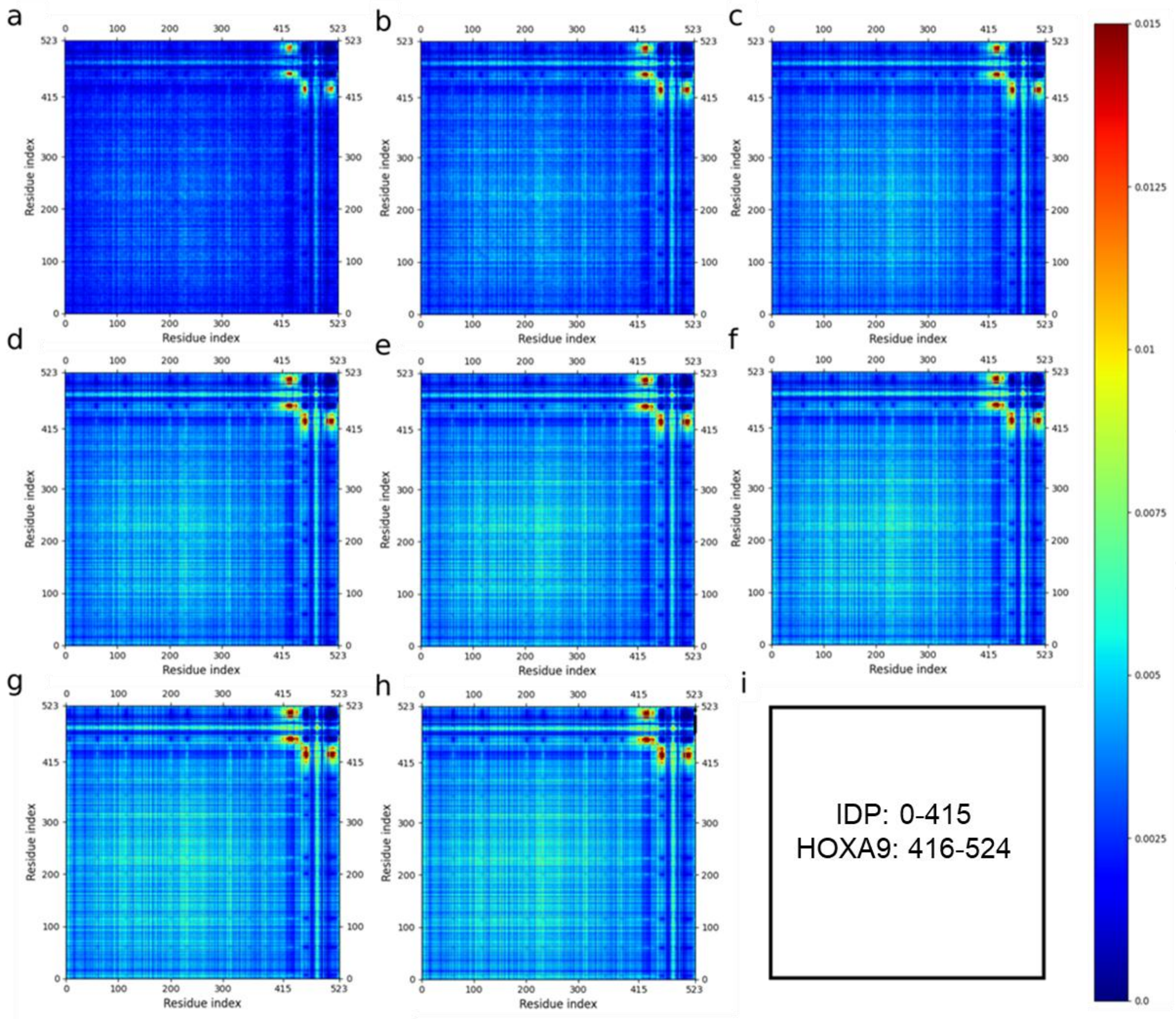

Supplementary Figure 19. Interchain contact map for clusters of different sizes. **a** 5, **b** 10, **c** 15, **d** 20, **e** 25, **f** 30, **g** 40, **h** 50 chains. **i** Indices of the amino acids making up each of the blocks/regions of NHA9. The contact probability per chain represents the mean number of contacts per frame between each pair of amino acids in the sequence, normalized by the mean cluster size (the number of NHA9 chains) as provided in Supplementary Table 3. Source data is provided at the data repository[6] (https://doi.org/10.17617/3.NIKDDY).

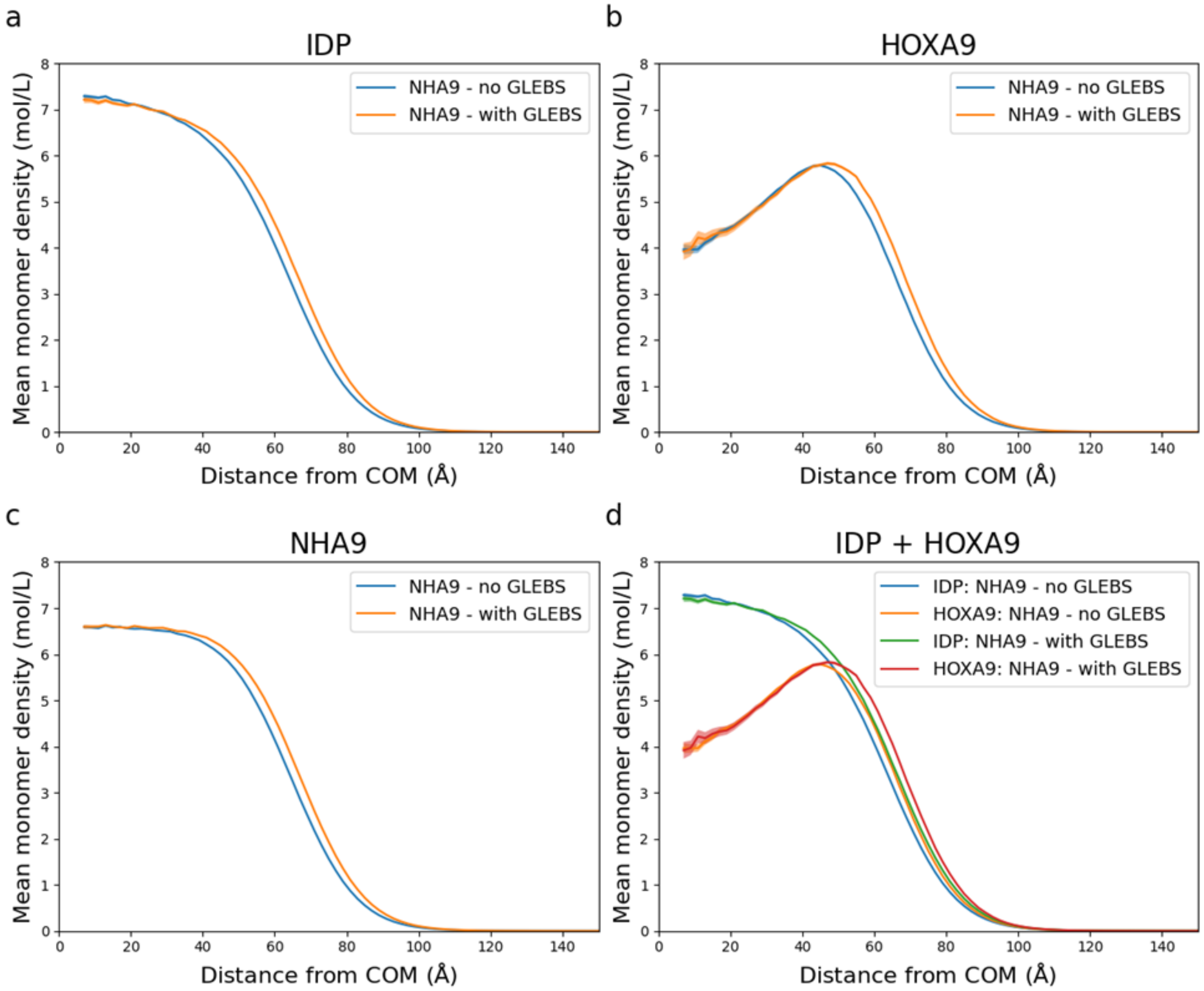

Supplementary Figure 20. Comparison of simulation results for NHA9 with and without the GLEBS domain. Simulations with 10 chains of NHA9 including the GLEBS domain (full-length NHA9) were performed. Units are moles of amino acids per liter. Both the intrinsically disordered protein (IDP) block (**a**) and the HOXA9 block (**b**) exhibited highly similar density profiles, as did the overall sequence (**c**), when comparing constructs with and without the GLEBS domain. **d** Overlay of the curves from panels (a) and (b) for direct comparison. Data are presented as mean values ± standard error of the mean (SEM). Error bars are shown as shaded regions, which in most cases are too small to be visible. The number of statistically independent samples for each point on the x-axis corresponds to the number of frames in the production run, listed in the last column of Supplementary Table 3. Source data are provided as a Source Data file.

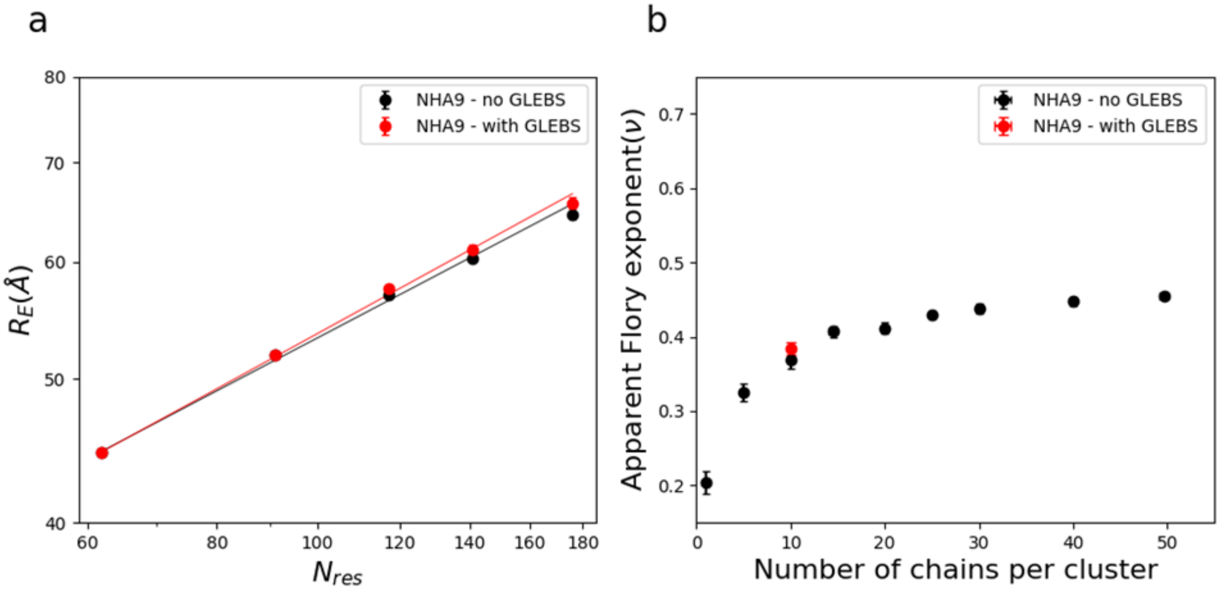

Supplementary Figure 21. R_E values and apparent Flory exponents of NHA9 with and without the GLEBS domain. Simulations of NHA9 including the GLEBS domain (full-length NHA9) with 10 chains were conducted. Black curves/dots represent NHA9 without the GLEBS domain and red curves/dots represent NHA9 with GLEBS domain. **a** Mean end-to-end distances (R_E) vs sequence distance (N_{res}) for clusters of 10 chains, showing minor residue-specific variation. The solid lines show scaling law fitting. Data are presented as mean values ± standard error of the mean (SEM). The number of samples varies for each point and is provided in Supplementary Table 4. **b** Apparent Flory exponent (ν) as a function of the number of chains in cluster. Data are presented as mean values ± the square root of the diagonal term of the covariance matrix of the fit corresponding to ν. A slight increase in ν is observed upon inclusion of the GLEBS domain, but the difference remains within error. Source data are provided as a Source Data file.

Supplementary Table 1. List of plasmids used in this study

Name [vector_gene(s)]	Promoters	In Figure
pQE_14His::TEV::NHA9$^{\Delta\text{GLEBS, A221TAG-S283Cys}}$	T5	Fig. 1-4, Supplementary Fig. 3-7
pQE_14His::TEV::NHA9$^{\Delta\text{GLEBS, A221TAG-S312Cys}}$	T5	Fig. 1-4, Supplementary Fig. 3-7
pQE_14His::TEV::NHA9$^{\Delta\text{GLEBS, A221TAG-S338Cys}}$	T5	Fig. 1-4, Supplementary Fig. 3-7
pQE_14His::TEV::NHA9$^{\Delta\text{GLEBS, A221TAG-S362Cys}}$	T5	Fig. 1-4, Supplementary Fig. 3-7
pQE_14His::TEV::NHA9$^{\Delta\text{GLEBS, A221TAG-S398Cys}}$	T5	Fig. 1-4, Supplementary Fig. 3-7
pQE_14His::TEV::NHA9$^{\Delta\text{GLEBS}}$	T5	Fig. 1-4, Supplementary Fig. 1-7
pQE_14His::TEV::FGdomain$^{\Delta\text{GLEBS}}$	T5	Supplementary Fig. 1
pBI_Flag::NHA9::HA<br>_Flag::NHA9::HA::P2AT2A::NHA9	pBI_CMV	Supplementary Fig. 10
pBI_Flag::NHA9$^{\text{A221TAG}}$::HA(boxB)<br>_Flag::NHA9::HA::P2AT2A::NHA9	pBI_CMV	Fig. 5, Supplementary Fig. 12
pBI_Flag::NHA9$^{\text{A221TAG-S283TAG}}$::HA(boxB)<br>_Flag::NHA9::HA::P2AT2A::NHA9	pBI_CMV	Fig. 5, Supplementary Fig. 12
pBI_Flag::NHA9$^{\text{A221TAG-S312TAG}}$::HA(boxB)<br>_Flag::NHA9::HA::P2AT2A::NHA9	pBI_CMV	Fig. 5, Supplementary Fig. 12
pBI_Flag::NHA9$^{\text{A221TAG-T353TAG}}$::HA(boxB)<br>_Flag::NHA9::HA::P2AT2A::NHA9	pBI_CMV	Fig. 5, Supplementary Fig. 12
pBI_Flag::NHA9$^{\text{A221TAG-S362TAG}}$::HA(boxB)<br>_Flag::NHA9::HA::P2AT2A::NHA9	pBI_CMV	Fig. 5, Supplementary Fig. 12
pBI_Flag::NHA9$^{\text{A221TAG-S98TAG}}$::HA(boxB)<br>_Flag::NHA9::HA::P2AT2A::NHA9	pBI_CMV	Fig. 5, Supplementary Fig. 12
pBI_Flag::NHA9$^{\Delta\text{GLEBS·A221TAG-S283TAG}}$::HA(boxB)<br>_Flag::NHA9$^{\Delta\text{GLEBS}}$::HA::P2AT2A::NHA9$^{\Delta\text{GLEBS}}$	pBI_CMV	Supplementary Fig. 13
pBI_Flag::NHA9$^{\Delta\text{GLEBS·A221TAG-S312TAG}}$::HA(boxB)<br>_Flag::NHA9 $^{\Delta\text{GLEBS}}$::HA::P2AT2A::NHA9$^{\Delta\text{GLEBS}}$	pBI_CMV	Supplementary Fig. 13
pBI_Flag::NHA9$^{\Delta\text{GLEBS·A221TAG-S362TAG}}$::HA(boxB)<br>_Flag::NHA9$^{\Delta\text{GLEBS}}$::HA::P2AT2A::NHA9$^{\Delta\text{GLEBS}}$	pBI_CMV	Supplementary Fig. 13
pcDNA3.1_mEGFP::4×GGS::NHA9	CMV	Fig. 5, Supplementary Fig. 11
pcDNA3.1_TOM20$_{1\text{-}70}$::FUS$_{1\text{-}478}$::V5::Myc::4xλ_{N22}::<br>NES::PylRS$^{\text{Y306A,Y384F}}$_U6-tRNA$^{\text{Pyl,CUA}}$	CMV, U6	Fig. 5, Supplementary Fig. 11, 12, 13

Supplementary Table 2. Amino acid sequence of proteins used in this study

Construct	Sequence
NHA9 (no GLEBS domain)	SGSMFNKSFGTPFGGGTGGFGTTSTFGQNTGFGTTSGGAFGTSAFGSSNNTGGLF<br>GNSQTKPGGLFGTSSFSQPATSTSTGFGFGTSTGTANTLFGTASTGTSLFSSQNNAF<br>AQNKPTGFGNFGTSTSSGGLFGTTNTTSNPFGSTSGSLFGPSSFTAAGPQNQVGA(<br>A221)GTTTGLFGSSPATSSATGLFSSSTTNSGFAYGQNKTAFGTSTTGFGTNPGGL<br>FGQQNQQTTS(S283)LFSKPFGQATTTQNTGFSFGNTSTIGQPS(S312)TNTMGLFG<br>VTQASQPGGLFGTATNTS(S338)TGTAFGTGTGLFGQTNTGFGAVGS(S362)TLFG<br>NNKLTTFGSSTTSAPSFGTTSGGLFGFGTNTS(S398)GNSIFGSKPAPGTLGTGLGA<br>GFGTALGAGQASLFGNNQPKIGGPLGTGAFGAPGFNTTTATLGFGAPQAPVVDRE<br>KQPSEGAFSENNAENESGGDKPPIDPNNPAANWLHARSTRKKRAPYTKHQTLELE<br>KEFLFNMYLTRDRRYEVARLLNLTERQVKIWFQNRRMKMKKINKDRAKDE
FG domain (no GLEBS domain)	SGSMFNKSFGTPFGGGTGGFGTTSTFGQNTGFGTTSGGAFGTSAFGSSNNTGGLF<br>GNSQTKPGGLFGTSSFSQPATSTSTGFGFGTSTGTANTLFGTASTGTSLFSSQNNAF<br>AQNKPTGFGNFGTSTSSGGLFGTTNTTSNPFGSTSGSLFGPSSFTAAGPQNQVGAG<br>TTTGLFGSSPATSSATGLFSSSTTNSGFAYGQNKTAFGTSTTGFGTNPGGLFGQQN<br>QQTTSLFSKPFGQATTTQNTGFSFGNTSTIGQPSTNTMGLFGVTQASQPGGLFGTA<br>TNTSTGTAFGTGTGLFGQTNTGFGAVGSTLFGNNKLTTFGSSTTSAPSFGTTSGGL<br>FGFGTNTSGNSIFGSKPAPGTLGTGLGAGFGTALGAGQASLFGNNQPKIGGPLGT<br>GAFGAPGFNTTTATLGFGAPQAPV
Flag::NHA9::HA::<br>P2AT2A::NHA9	DYKDDDDKMFNKSFGTPFGGGTGGFGTTSTFGQNTGFGTTSGGAFGTSA<br>FGSSNNTGGLFGNSQTKPGGLFGTSSFSQPATSTSTGFGFGTSTGTANTLF<br>GTASTGTSLFSSQNNAFAQNKPTGFGNFGTSTSSGGLFGTTNTTSNPFGST<br>SGSLFGPSSFTAAPTGTTIKFNPPTGTDTMVKAGVSTNISTKHQCITAMKE<br>YESKSLEELRLEDYQANRKGPQNQVGAGTTTGLFGSSPATSSATGLFSSST<br>TNSGFAYGQNKTAFGTSTTGFGTNPGGLFGQQNQQTTSLFSKPFGQATTT<br>QNTGFSFGNTSTIGQPSTNTMGLFGVTQASQPGGLFGTATNTSTGTAFGT<br>GTGLFGQTNTGFGAVGSTLFGNNKLTTFGSSTTSAPSFGTTSGGLFGFGTN<br>TSGNSIFGSKPAPGTLGTGLGAGFGTALGAGQASLFGNNQPKIGGPLGTG<br>AFGAPGFNTTTATLGFGAPQAPVVDREKQPSEGAFSENNAENESGGDKPP<br>IDPNNPAANWLHARSTRKKRCPYTKHQTLELEKEFLFNMYLTRDRRYEV<br>ARLLNLTERQVKIWFQNRRMKMKKINKDRAKDEGGSGGSYPYDVPDYA<br>TGGSGATNFSLLKQAGDVEENPGPGSGEGRGSLLTCGDVEENPGPLQMF<br>NKSFGTPFGGGTGGFGTTSTFGQNTGFGTTSGGAFGTSAFGSSNNTGGLF<br>GNSQTKPGGLFGTSSFSQPATSTSTGFGFGTSTGTANTLFGTASTGTSLFSS<br>QNNAFAQNKPTGFGNFGTSTSSGGLFGTTNTTSNPFGSTSGSLFGPSSFTA<br>APTGTTIKFNPPTGTDTMVKAGVSTNISTKHQCITAMKEYESKSLEELRLE<br>DYQANRKGPQNQVGAGTTTGLFGSSPATSSATGLFSSSTTNSGFAYGQNK<br>TAFGTSTTGFGTNPGGLFGQQNQQTTSLFSKPFGQATTTQNTGFSFGNTST<br>IGQPSTNTMGLFGVTQASQPGGLFGTATNTSTGTAFGTGTGLFGQTNTGF<br>GAVGSTLFGNNKLTTFGSSTTSAPSFGTTSGGLFGFGTNTSGNSIFGSKPAP<br>GTLGTGLGAGFGTALGAGQASLFGNNQPKIGGPLGTGAFGAPGFNTTTAT<br>LGFGAPQAPVVDREKQPSEGAFSENNAENESGGDKPPIDPNNPAANWLH<br>ARSTRKKRCPYTKHQTLELEKEFLFNMYLTRDRRYEVARLLNLTERQVKI<br>WFQNRRMKMKKINKDRAKDE

mEGFP::4×GGS:: NHA9	VSKGEELFTGVVPILVELDGDVNGHKFSVSGEGEGDATYGKLTLKFICTT<br>GKLPVPWPTLVTTLTYGVQCFSRYPDHMKQHDFFKSAMPEGYVQERTIF<br>FKDDGNYKTRAEVKFEGDTLVNRIELKGIDFKEDGNILGHKLEYNYNSHN<br>VYIMADKQKNGIKVNFKIRHNIEDGSVQLADHYQQNTPIGDGPVLLPDNH<br>YLSTQSKLSKDPNEKRDHMVLLEFVTAAGITLGMDELYKGGSGGSGGSG<br>GSMFNKSFGTPFGGGTGGFGTTSTFGQNTGFGTTSGGAFGTSAFGSSNNT<br>GGLFGNSQTKPGGLFGTSSFSQPATSTSTGFGFGTSTGTANTLFGTASTGT<br>SLFSSQNNAFAQNKPTGFGNFGTSTSSGGLFGTTNTTSNPFGSTSGSLFGPS<br>SFTAAPTGTTIKFNPPTGTDTMVKAGVSTNISTKHQCITAMKEYESKSLEE<br>LRLEDYQANRKGPQNQVGAGTTTGLFGSSPATSSATGLFSSSTTNSGFAY<br>GQNKTAFGTSTTGFGTNPGGLFGQQNQQTTSLFSKPFGQATTTQNTGFSF<br>GNTSTIGQPSTNTMGLFGVTQASQPGGLFGTATNTSTGTAFGTGTGLFGQ<br>TNTGFGAVGSTLFGNNKLTTFGSSTTSAPSFGTTSGGLFGFGTNTSGNSIFG<br>SKPAPGTLGTGLGAGFGTALGAGQASLFGNNQPKIGGPLGTGAFGAPGFN<br>TTTATLGFGAPQAPVVDREKQPSEGAFSENNAENESGGDKPPIDPNNPAA<br>NWLHARSTRKKRCPYTKHQTLELEKEFLFNMYLTRDRRYEVARLLNLTE<br>RQVKIWFQNRRMKMKKINKDRAKDE

Supplementary Table 3. Simulation details and mean number of chains in clusters. The mean number of chains in each cluster is given as the mean value ± standard deviation (SD).

Sequence	N. chains	N. particles	Volume fraction	Mean N. of chains in cluster	Box side length (L x L x L)	Frames in production run
NHA9 no GLEBS	1	33536	0.0002	-	2400.00 Å	7099
NHA9 no GLEBS	5	2620	0.001	5	622.87 Å	8563
NHA9 no GLEBS	10	5240	0.001	10	784.77 Å	7274
NHA9 no GLEBS	15	7860	0.001	14.47 ± 0.50	898.33 Å	8559
NHA9 no GLEBS	20	10480	0.001	20	988.74 Å	7880
NHA9 no GLEBS	25	13100	0.001	25	1065.09 Å	7888
NHA9 no GLEBS	30	15720	0.001	30	1131.83 Å	7673
NHA9 no GLEBS	40	20960	0.001	40	1245.74 Å	7090
NHA9 no GLEBS	50	26200	0.001	49.65 ± 0.47	1341.93 Å	8027
NHA9 with GLEBS	10	5810	0.005	10	476.92 Å	2689

Supplementary Table 4. Number of independent samples $(n_{eff}.n_{chains})$ used to calculate the error bars (SEM) for mean distances between amino acids in Figure 6a for each FRET pair. They are computed as described in methods and correspond to the effective number of statistically independent values obtained.

N. chains / sequence	A221-S283	A221-312	A221-S338	A221-S362	A221-S398
1 / NHA9 no GLEBS	454336	454336	454336	454336	454336
5 / NHA9 no GLEBS	42820	42820	33296	13494	10970
10 / NHA9 no GLEBS	72750	34107	11920	9653	8129
15 / NHA9 no GLEBS	123873	25421	16555	12922	10353
20 / NHA9 no GLEBS	157620	23767	16588	12909	9265
25 / NHA9 no GLEBS	197225	25433	16522	13398	10146
30 / NHA9 no GLEBS	230220	26779	16836	12919	8965
40 / NHA9 no GLEBS	277322	28551	18534	13058	10093
50 / NHA9 no GLEBS	363904	47030	30138	21046	14460
10 / NHA9 with GLEBS	26900	9931	4536	3309	2310

Reference

1. Lakowicz, J. R. *Principles of Fluorescence Spectroscopy*. (Springer, 2006).

2. Reinkemeier, C. D., Girona, G. E. & Lemke, E. A. Designer membraneless organelles enable codon reassignment of selected mRNAs in eukaryotes. *Science* **363**, eaaw2644 (2019).

3. Reinkemeier, C. D. & Lemke, E. A. Dual film-like organelles enable spatial separation of orthogonal eukaryotic translation. *Cell* **184**, 4886-4903.e21 (2021).

4. Yu, M. *et al.* Visualizing the disordered nuclear transport machinery in situ. *Nature* **617**, 162–169 (2023).

5. Stukowski, A. Visualization and analysis of atomistic simulation data with OVITO–the Open Visualization Tool. *Modelling Simul. Mater. Sci. Eng.* **18**, 015012 (2010).

6. Dillenburg, R. *et al.* Simulation data: Differential conformational expansion of NUP98-HOXA9 oncoprotein from nanosized assemblies to macrophases. *Edmond* https://doi.org/10.17617/3.NIKDDY (2025).
