# Supplementary material for: Differential conformational expansion of NUP98-HOXA9 oncoprotein from nanosized assemblies to macrophases
Source: Nat Commun. 2025 Nov 18;16:10117. doi: 10.1038/s41467-025-66327-1 (PMC12627784; doi:10.1038/s41467-025-66327-1)
Supplement: Supplementary file 2 — Reporting Summary [file 41467_2025_66327_MOESM2_ESM.pdf]

Corresponding author(s): Edward A. Lemke & Martin Girard

Last updated by author(s): Oct 25, 2025

Reporting Summary

Nature Portfolio wishes to improve the reproducibility of the work that we publish. This form provides structure for consistency and transparency in reporting. For further information on Nature Portfolio policies, see our Editorial Policies and the Editorial Policy Checklist.

Statistics

For all statistical analyses, confirm that the following items are present in the figure legend, table legend, main text, or Methods section.

n/a	Confirmed	
☐	☒	The exact sample size (n) for each experimental group/condition, given as a discrete number and unit of measurement
☐	☒	A statement on whether measurements were taken from distinct samples or whether the same sample was measured repeatedly
☒	☐	The statistical test(s) used AND whether they are one- or two-sided<br>*Only common tests should be described solely by name; describe more complex techniques in the Methods section.*
☐	☒	A description of all covariates tested
☐	☒	A description of any assumptions or corrections, such as tests of normality and adjustment for multiple comparisons
☐	☒	A full description of the statistical parameters including central tendency (e.g. means) or other basic estimates (e.g. regression coefficient) AND variation (e.g. standard deviation) or associated estimates of uncertainty (e.g. confidence intervals)
☒	☐	For null hypothesis testing, the test statistic (e.g. F, t, r) with confidence intervals, effect sizes, degrees of freedom and P value noted<br>*Give P values as exact values whenever suitable.*
☒	☐	For Bayesian analysis, information on the choice of priors and Markov chain Monte Carlo settings
☒	☐	For hierarchical and complex designs, identification of the appropriate level for tests and full reporting of outcomes
☒	☐	Estimates of effect sizes (e.g. Cohen's d, Pearson's r), indicating how they were calculated

Our web collection on statistics for biologists contains articles on many of the points above.

Software and code

Policy information about availability of computer code

Data collection	Symphotime v2.6; Hoomd-Blue v5.0.1;gsd - v2.9.0.
Data analysis	Symphotime v2.6, PAM-PIE(https://gitlab.com/PAM-PIE/PAM), Matlab R2021a, Fiji;gsd - v2.9.0;freud - v2.13.2; OVITO.<br>Codes related to simulations and analysis are available at https://gitlab.mpcdf.mpg.de/dillenburgr/nha9.

For manuscripts utilizing custom algorithms or software that are central to the research but not yet described in published literature, software must be made available to editors and reviewers. We strongly encourage code deposition in a community repository (e.g. GitHub). See the Nature Portfolio guidelines for submitting code & software for further information.

Data

Policy information about availability of data

All manuscripts must include a data availability statement. This statement should provide the following information, where applicable:

- Accession codes, unique identifiers, or web links for publicly available datasets
- A description of any restrictions on data availability
- For clinical datasets or third party data, please ensure that the statement adheres to our policy

All trajectories from MD simulations and a corresponding analysis data of the contact maps corresponding to Supplementary Figure 19 are deposited on the Edmond repository (https://doi.org/10.17617/3.NIKDDY) [Dillenburg, Rodrigo, Hao Ruan, Elnaz Hosseini, Sina Wittmann, Martin Girard, and Edward Lemke. 2025. "Simulation Data: Differential Conformational Expansion of NUP98-HOXA9 Oncoprotein from Nanosized Assemblies to Macrophases." Edmond. https://doi.org/doi:10.17617/3.NIKDDY.]. All other data are available in the main text or Supplementary Information. Source data are provided with this paper.

Research involving human participants, their data, or biological material

Policy information about studies with human participants or human data. See also policy information about sex, gender (identity/presentation), and sexual orientation and race, ethnicity and racism.

Reporting on sex and gender	NA
Reporting on race, ethnicity, or other socially relevant groupings	NA
Population characteristics	NA
Recruitment	NA
Ethics oversight	NA

Note that full information on the approval of the study protocol must also be provided in the manuscript.

Field-specific reporting

Please select the one below that is the best fit for your research. If you are not sure, read the appropriate sections before making your selection.

[x] Life sciences   [ ] Behavioural & social sciences   [ ] Ecological, evolutionary & environmental sciences

For a reference copy of the document with all sections, see nature.com/documents/nr-reporting-summary-flat.pdf

Life sciences study design

All studies must disclose on these points even when the disclosure is negative.

Sample size	The sample size (~30 cells per mutant) in FLIM-FRET measurements was chosen so that the total photon numbers are sufficient enough for robust fitting of fluorescence lifetime decays across all five mutants.
Data exclusions	For each mutant, cells with phasor values outside the mean ± 3 standard deviations were excluded from further analysis
Replication	The acceptor photobleaching assay for assessing both intermolecular and intramolecular FRET was performed independently five times, yielding consistent results. Immunofluorescence imaging was repeated three times independently, with all replication attempts proving successful
Randomization	Cells were seeded, transfected, and labelled in imaging dishes. The red fluorescent channel was used to identify cells exhibiting a fluorescently labeled nuclear condensates. Following image acquisition, a threshold (as detailed in the Methods) was applied to exclude cells with excessively high expression levels from analysis. No additional randomization was performed.
Blinding	The investigators were blinded to group allocation during data collection and analysis.

Reporting for specific materials, systems and methods

We require information from authors about some types of materials, experimental systems and methods used in many studies. Here, indicate whether each material, system or method listed is relevant to your study. If you are not sure if a list item applies to your research, read the appropriate section before selecting a response.

Materials & experimental systems

n/a	Involved in the study
[ ]	[x] Antibodies
[ ]	[x] Eukaryotic cell lines
[x]	[ ] Palaeontology and archaeology
[x]	[ ] Animals and other organisms
[x]	[ ] Clinical data
[x]	[ ] Dual use research of concern
[x]	[ ] Plants

Methods

n/a	Involved in the study
[x]	[ ] ChIP-seq
[x]	[ ] Flow cytometry
[x]	[ ] MRI-based neuroimaging

Antibodies

Antibodies used

Anti-H3K27Ac(Abcam, ab4729), Anti-H3K9me3(Abcam, ab8898), anti-HA (Sigma-Aldrich, H9658), second antibody (anti-rabbit, Alexa Fluor 488, thermoFisher, A-11008), secondary antibody(anti-mouse HRP-conjugated, Jackson ImmunoResearch, 715-035-150)

Validation

Antibodies were validated by the manufactures.
For Anti-H3K27Ac, https://www.abcam.com/en-us/products/primary-antibodies/histone-h3-acetyl-k27-antibody-chip-grade-ab4729.
For Anti-H3K9me3, https://www.abcam.com/en-us/products/primary-antibodies/histone-h3-tri-methyl-k9-antibody-chip-grade-ab8898.
For anti-HA, https://www.sigmaaldrich.com/DE/en/product/sigma/h9658?cm_sp=Insite-_-prodRecCold_xorders-_-prodRecCold2-1.
For secondary antibody-Alexa488, https://www.thermofisher.com/antibody/product/Goat-anti-Rabbit-IgG-H-L-Cross-Adsorbed-Secondary-Antibody-Polyclonal/A-11008.
For anti-mouse HRP-conjugated (Jackson ImmunoResearch, 715-035-150), https://www.jacksonimmuno.com/catalog/products/715-035-150

Eukaryotic cell lines

Policy information about cell lines and Sex and Gender in Research

Cell line source(s)

HelaK (RRID: CVCL_1922)

Authentication

A gift from Martin Beck's laboratory

Mycoplasma contamination

All cell lines were regularly tested for mycoplasma contamination, with negative results.

Commonly misidentified lines
(See ICLAC register)

No misindentified lines were used

Plants

Seed stocks

Report on the source of all seed stocks or other plant material used. If applicable, state the seed stock centre and catalogue number. If plant specimens were collected from the field, describe the collection location, date and sampling procedures.

Novel plant genotypes

Describe the methods by which all novel plant genotypes were produced. This includes those generated by transgenic approaches, gene editing, chemical/radiation-based mutagenesis and hybridization. For transgenic lines, describe the transformation method, the number of independent lines analyzed and the generation upon which experiments were performed. For gene-edited lines, describe the editor used, the endogenous sequence targeted for editing, the targeting guide RNA sequence (if applicable) and how the editor was applied.

Authentication

Describe any authentication procedures for each seed stock used or novel genotype generated. Describe any experiments used to assess the effect of a mutation and, where applicable, how potential secondary effects (e.g. second site T-DNA insertions, mosiacism, off-target gene editing) were examined.
